# Tratamento Crônico com *Panax ginseng* e *Angelica keiskei* Reduz a Pressão Arterial e Melhora a Função Endotelial em Ratas Ovariectomizadas

**DOI:** 10.36660/abc.20240685

**Published:** 2025-08-20

**Authors:** Nayara Formenton da Silva, Luis Henrique Oliveira de Moraes, Camila Pereira Sabadini, Rita Cristina Cotta Alcântara, Patrícia Corrêa Dias, Gerson Jhonatan Rodrigues

**Affiliations:** 1 Universidade Federal de São Carlos São Carlos SP Brasil Universidade Federal de São Carlos (UFSCar), São Carlos, SP – Brasil

**Keywords:** Ovariectomia, Menopausa, Hipertensão, Fitoterapia, Panax

## Abstract

**Fundamento:**

A menopausa é um processo fisiológico natural que pode afetar diversos sistemas e órgãos, frequentemente levando ao aumento da pressão arterial. A combinação de *Panax ginseng* e *Angelica keiskei* (Pg + Ak) é uma formulação conhecida por melhorar a função vascular.

**Objetivo:**

Este estudo teve como objetivo avaliar os efeitos *in vivo* dessas plantas combinadas sobre os parâmetros cardiovasculares em ratas em menopausa induzida por ovariectomia.

**Métodos:**

Vinte e quatro ratas Wistar com 70 dias de vida foram distribuídas aleatoriamente em três grupos: falso-operadas (Sham), ovariectomizadas (OVX) e ovariectomizadas tratadas com Pg + Ak (OVX + Pg + Ak). As ratas do grupo tratado com Pg + Ak receberam injeção intraperitoneal de 100 mg/kg/dia por duas semanas. A pressão arterial foi medida por pletismografia de cauda, e a função endotelial foi avaliada por meio de testes de reatividade vascular.

**Resultados:**

Ao final do tratamento, a pressão arterial média no grupo Sham foi menor do que no grupo controle (OVX), e o tratamento com Pg + Ak melhorou a disfunção endotelial causada pela ovariectomia. Isso ficou evidente na restauração da vasodilatação dependente do endotélio nos anéis aórticos das ratas OVX tratadas com Pg + Ak, em comparação com as ratas OVX não tratadas.

**Conclusões:**

Nossos achados sugerem que a combinação Pg + Ak reduz efetivamente a pressão arterial e reverte a disfunção endotelial em ratas ovariectomizadas.

## Graphical abstract

**Figura Central:**
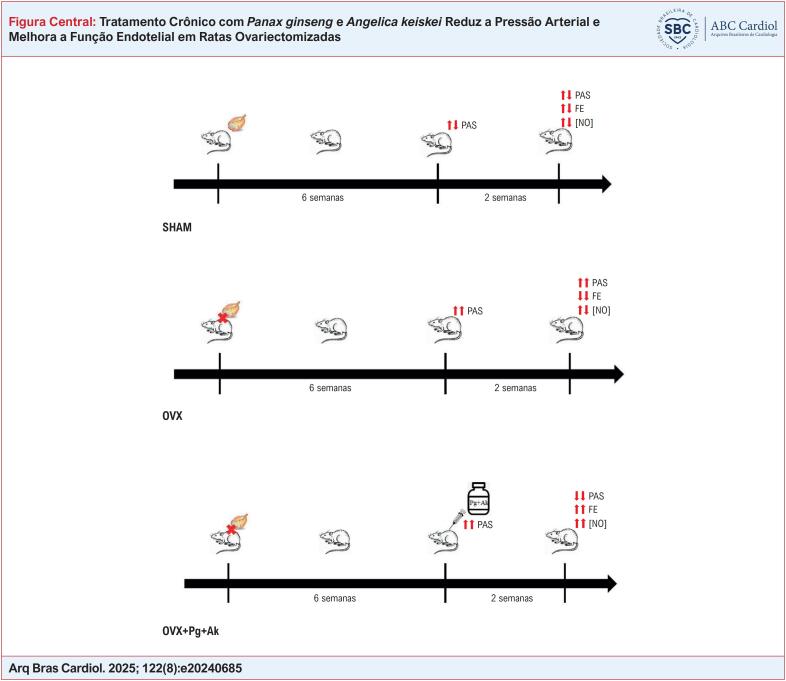
Tratamento Crônico com Panax ginseng e Angelica keiskei Reduz a Pressão Arterial e Melhora a Função Endotelial em Ratas Ovariectomizadas

Representação cronológica de três grupos experimentais, A: SHAM (SH), B: ovariectomizada (OVX) e C: ovariectomizada tratada com Panax ginseng + Angelica keiskei (OVX+Pg+Ak). PAS: pressão arterial sistólica; FE: função endothelial; [NO]: concentração sérica. A seta para cima (↑) indica aumento, a seta para baixo (↓) indica diminuição e as setas para cima e para baixo (↑↓) indicam nenhuma alteração.

## Introdução

A menopausa é um processo fisiológico natural caracterizado pela cessação permanente da menstruação por 12 meses, geralmente relacionado à deficiência de estrogênio associada à idade e não vinculada a patologias. A idade mediana da menopausa é em torno de 51 anos. Embora a maioria das mulheres apresente sintomas vasomotores, a menopausa também afeta diversos sistemas do corpo, incluindo os sistemas urogenital, psicogênico e cardiovascular.^1 - 4^ A redução dos níveis de estrogênio prejudica o eixo hipotálamo-hipófise-ovário, levando à falha no desenvolvimento endometrial, o que resulta em ciclos menstruais irregulares que, por fim, cessam completamente.^5^

O estrogênio desempenha um papel crucial na manutenção da função endotelial ao aumentar a síntese de óxido nítrico (NO) no endotélio vascular. O NO difunde-se nas células musculares lisas vasculares, promovendo o relaxamento – um processo conhecido como vasodilatação dependente do endotélio (VDE). O estrogênio também contribui para a manutenção da função endotelial ao reduzir a síntese de endotelina-1, um potente vasoconstritor, nas células endoteliais. Durante a menopausa, a VDE diminui, enquanto a síntese de Endotelina-1 aumenta, ambos os fatores contribuindo para maior vasoconstrição.^6^

Além disso, a deficiência de estrogênio pode afetar diversos órgãos e sistemas, incluindo o sistema cardiovascular, aumentando o risco de doenças como a hipertensão. Segundo dados do *Global Burden of Disease* , a hipertensão é o principal fator de risco mortal entre mulheres em todo o mundo.^7^ A incidência de doenças cardiovasculares em mulheres pós-menopáusicas é de duas a seis vezes maior do que em mulheres pré-menopáusicas da mesma faixa etária.^8^ Para o manejo desses sintomas, a terapia de reposição hormonal (TRH) é frequentemente recomendada como forma de mitigar a deficiência de estrogênio, melhorando assim a qualidade de vida nos aspectos físicos, emocionais e sexuais.^9^ No entanto, devido aos efeitos colaterais conhecidos e às potenciais contraindicações da TRH, terapias botânicas alternativas têm sido cada vez mais reconhecidas como benéficas para essa população.^10^

Uma das espécies de ginseng mais amplamente utilizadas atualmente é o *Panax ginseng* , cultivado principalmente na Coreia e na China, responsável por uma parcela significativa da produção global de ginseng.^11^ Os principais compostos ativos do *Panax ginseng* são os ginsenosídeos, que são saponinas triterpênicas. Grande parte das pesquisas sobre as propriedades farmacológicas e medicinais do *Panax ginseng* concentra-se nos ginsenosídeos, incluindo Rb1, Rg1, Rg3, Re e Rd.^12^

O ginseng demonstrou diversas atividades biológicas, incluindo propriedades anticancerígenas,^13 , 14^ antidiabéticos,^15 , 16^ e benefícios cardiovasculares, como auxiliar no tratamento da hipertensão.^17 - 19^ Os ginsenosídeos Rb1 e Rg1 apresentam efeitos anti-inflamatórios ao inibir a produção de antioxidantes e promover a síntese de NO^20 , 21^ Além disso, esses ginsenosídeos possuem propriedades antioxidantes, prevenindo a produção de espécies reativas de oxigênio (EROs) por meio da estimulação do NO. O ginsenosídeo Rb1 e outros ginsenosídeos também combatem a disfunção endotelial ao ativar a via de sinalização SIRT1/AMPK, inibir a produção de EROs, ativar o receptor de estrogênio beta (ER-β) e aumentar a expressão da enzima superóxido dismutase (SOD).^23 , 24^

*Angelica keiskei* , comumente conhecida como *ashitaba* ou "folha do amanhã", é uma planta perene resistente, nativa da costa pacífica do Japão.^25^ Essa planta tem ganhado atenção por suas propriedades medicinais, incluindo efeitos antioxidantes, anti-inflamatórios, hipoglicemiantes, antimicrobianos, antitumorais, hipotensivos, antifibróticos, laxativos, estimulantes e galactagogos.^25 - 29^ Embora os componentes ativos específicos da *Angelica keiskei* ainda não tenham sido identificados, mais de 100 compostos já foram isolados dela, incluindo chalconas, cumarinas e flavanonas.^29^

Um produto conhecido como Mitochondrin® combina *Panax ginseng* e *Angelica keiskei* , apresentando uma tripla padronização de 8% de chalconas, 10% de flavanonas e 0,9% de ginsenosídeos Rb1, Rg1 e Rg3, proporcionando uma ação multialvo em diversos mecanismos epigenéticos. Esses componentes promovem a biogênese mitocondrial, essencial para a produção de energia e envelhecimento saudável, além de estimular o gasto calórico, reduzir o armazenamento de gordura e minimizar a inflamação por meio de efeitos antioxidantes.^30^

Essas plantas são amplamente utilizadas na medicina tradicional chinesa por seus benefícios fisiológicos, que incluem a redução de radicais livres,^31 , 32^ controle da inflamação,^33 , 34^ regulação da coagulação,^35^ promoção da produção de NO,^36^ melhora do metabolismo da glicose,^37^ e redução da disfunção mitocondrial.^23 , 38^ As terapias complementares têm ganhado destaque como uma opção segura para o manejo dos sintomas da menopausa. Neste estudo, investigamos se a combinação única de *Panax ginseng* e *Angelica keiskei* pode melhorar os parâmetros vasculares em ratas ovariectomizadas.

## Materiais e Métodos

### Animais e delineamento experimental

Vinte e quatro ratas Wistar fêmeas, com peso entre 200 g e 250 g, foram divididas aleatoriamente (cada animal recebeu um número e os grupos foram determinados por meio de um gerador de números aleatórios, evitando viés de seleção) em três grupos: ovariectomizadas (OVX) (n = 8), ovariectomizadas tratadas com extrato padronizado de *Panax ginseng* + *Angelica keiskei* (OVX + Pg + Ak) (n = 8) e falso-operadas (Sham) (n = 8). Todos os procedimentos com as ratas descritos neste trabalho foram realizados após uma semana de aclimatação. Os animais foram mantidos em ciclo claro-escuro com alimento e água disponíveis *ad libitum* até o dia do experimento. Todos os animais foram pesados a cada duas semanas.

### Indução da menopausa

Todos os animais dos grupos OVX foram submetidos à ovariectomia na 12ª semana de vida, devido à boa resposta à cirurgia evidenciada na literatura.^38^ A técnica utilizada seguiu o protocolo de Zarrow,^39^ e a anestesia foi realizada com uma associação de 13 mg/kg de xilazina e 33 mg/kg de base de cetamina por administração parenteral (intramuscular). Primeiramente, uma pequena incisão bilateral (1,0 – 1,5 cm) foi feita através da pele e da camada muscular, utilizando tesoura e pinça, aproximadamente 1 cm abaixo da última costela, perpendicular ao corpo do animal.

Para o procedimento, a cavidade peritoneal foi aberta, os ovários foram expostos e removidos, e uma sutura de náilon foi realizada logo abaixo da fímbria. Após a remoção dos ovários, uma incisão foi feita na musculatura e na pele com o fio de náilon. Os animais do grupo falso-operado (sham) foram submetidos ao mesmo procedimento cirúrgico, com os ovários sendo apenas exteriorizados da cavidade abdominal e depois recolocados, sem serem removidos. Após a cirurgia, foi respeitado um período de duas semanas de recuperação antes do início dos procedimentos com o protocolo de administração do extrato padronizado.

### Medida da pressão arterial

Ao longo do tratamento, a pressão arterial sistólica dos animais foi medida por pletismografia em animmais não anestasiados de cauda — antes da indução da menopausa, seis semanas após a indução e após o tratamento. As ratas foram colocadas em um aparato de contenção e aclimatação, em um ambiente calmo e silencioso, por uma hora. Esse procedimento foi repetido algumas vezes para que os ratos se familiarizassem com o teste.

O pletismógrafo possui uma braçadeira de borracha e um sensor fotoelétrico de pulso que foi colocado ao redor da cauda do animal (modelo Power Lab 8/35, AD Instruments, Pty Ltda, Colorado Springs, CO). Foram realizadas três medições consecutivas, considerando a média aritmética desses resultados como o valor da pressão.

### Administração dos extratos padronizados de *Panax ginseng* + *Angelica keiskei*

Os animais dos grupos que receberam o tratamento com extrato padronizado foram tratados com 100 mg/kg/dia por duas semanas, e o extrato padronizado foi administrado por via intraperitoneal.²³ Todos os animais foram pesados para que a dose do extrato padronizado fosse adequada. Os animais dos grupos que não receberam o tratamento com o extrato padronizado foram manuseados com a mesma frequência que os tratados. Os extratos padronizados da associação de Panax ginseng + Angelica keiskei (1:1) compõem a formulação de um produto registrado sob o nome comercial Mitochondrin®. Este produto apresenta tripla padronização: 8% de chalconas, 10% de flavanonas e 0,9% de ginsenosídeos Rb1, Rg1 e Rg3. Os extratos foram solubilizados em solução fisiológica (cloreto de sódio a 0,9%) imediatamente antes da administração.

### Eutanásia e coleta de material biológico

Os animais, anestesiados com isofluorano, foram sacrificados por decapitação e alguns tecidos foram coletados para análises posteriores. A artéria torácica da aorta foi removida, isolada e cortada em anéis de aproximadamente 4 mm de comprimento, sendo mantida em solução de Krebs para o experimento de reatividade vascular. O útero foi coletado e fotografado para análises adicionais. O sucesso da cirurgia foi verificado por meio da atrofia uterina observada nos grupos ovariectomizadas, em comparação ao grupo falso-operado, conforme pode ser verificado na Figura Central e no material suplementar ( Imagem 1A ). O sangue foi coletado em tubos Falcon logo após a eutanásia, para posterior obtenção de alíquotas de soro destinadas a testes bioquímicos.

### Reatividade vascular

Os anéis aórticos dissecados após a eutanásia foram colocados em um banho de órgão isolado contendo 5 mL de solução de Krebs a 37 °C, pH 7,4, borbulhada continuamente com 95% de O_2_ e 5% de CO_2_, em um miógrafo isométrico (modelo Mulvany-Halpern 610 DMT-USA, Marietta, GA) e registrados por um sistema de aquisição de dados PowerLab8/SP (ADInstruments Pty Ltd., Colorado Springs, CO).

Primeiramente, os anéis aórticos foram submetidos a uma tensão de 1,5 g por trinta minutos para permitir a estabilização. Em seguida, a integridade endotelial foi avaliada utilizando a EC50 da fenilefrina (0,1 *µ* mol/L) para contrair o vaso, seguida do relaxamento induzido por 1 *µ* mol/L de acetilcolina. Os anéis foram descartados quando o relaxamento foi inferior a 80%, conforme o padrão estabelecido para ratos hipertensos com disfunção endotelial.^40^

Os anéis aórticos íntegros foram submetidos a uma segunda contração com 0,1 *µ* mol/L de fenilefrina e, então, foram construídas curvas concentração-resposta à acetilcolina (0,1 nmol/L a 0,1 mmol/L). A potência (pD_2_) e o efeito relaxante máximo foram avaliados.

### Medida do óxido nítrico

Para a avaliação dos níveis de NO, foi medida a concentração sérica dos produtos estáveis do NO, nitrito (NO_2_^–^) e nitrato (NO_3_^–^), conhecidos como NOx, conforme descrito anteriormente,^41^ utilizando o NO Analyzer 280i (Sievers, Boulder, CO, EUA).

### Análise estatística

As análises estatísticas dos resultados foram realizadas utilizando o software GraphPad Prism (versão 8.0.1). A distribuição dos dados foi avaliada quanto à normalidade por meio do teste de Shapiro-Wilk. Como os dados apresentaram distribuição normal, eles foram apresentados como média ± erro padrão da média (EP). As comparações entre todos os grupos foram realizadas utilizando ANOVA de uma via com o pós-teste de Newman-Keuls. Adotou-se um nível de significância de 95% (α = 0,05) para todas as análises estatísticas, garantindo que a probabilidade de erro tipo I permanecesse dentro de um limite aceitável para inferências robustas e confiáveis.

## Resultados

**Figura 1:**
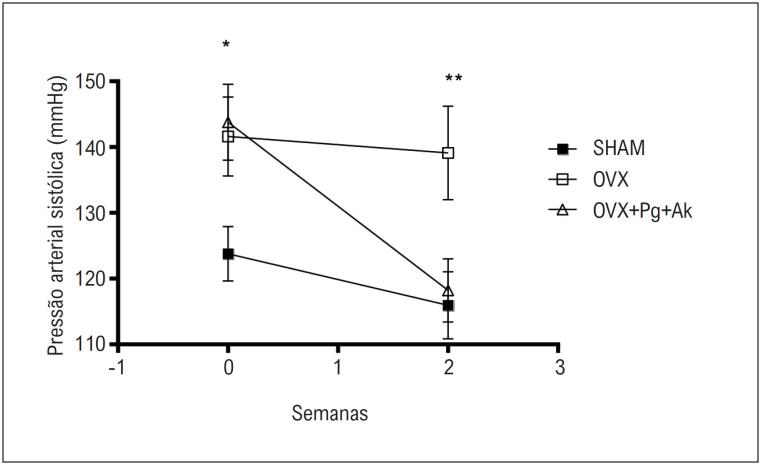
Pressão arterial sistólica antes e após o tratamento de duas semanas. Cada ponto representa a média e o erro padrão da média (EP) dos dados obtidos a partir de determinações independentes. *Indica diferença (p < 0,05) nos valores de pressão arterial sistólica entre Sham vs. OVX e Sham vs. OVX + Pg + Ak antes do tratamento com Pg + Ak (tempo = 0). Indica a diferença (p < 0,05) entre os valores de pressão arterial sistólica dos grupos OVX vs. OVX + Pg + Ak após duas semanas de tratamento com Pg + Ak (tempo = 2); OVX: ratas ovariectomizadas; OVX + Pg + Ak: ratas ovariectomizadas tratadas com extrato padronizado de Panax ginseng + Angelica keiskei.

A ovariectomia induziu elevação da pressão arterial sistólica, como pode ser observado no grupo OVX (141,62 ± 6,0 mmHg, n = 8) e no grupo OVX + Pg + Ak antes do tratamento (143,77 ± 5,79 mmHg, n = 8), em comparação ao grupo Sham (123,77 ± 4,16 mmHg, n = 8). O tratamento por duas semanas com a associação de *Panax ginseng* + *Angelica keiskei* induziu redução da pressão arterial sistólica em ratas OVX (OVX + Pg + Ak: 118,22 ± 4,8 mmHg, n = 8), em comparação ao grupo OVX sem tratamento (139,12 ± 7,1 mmHg, n = 8) ( Figura 1 ).

**Figura 2:**
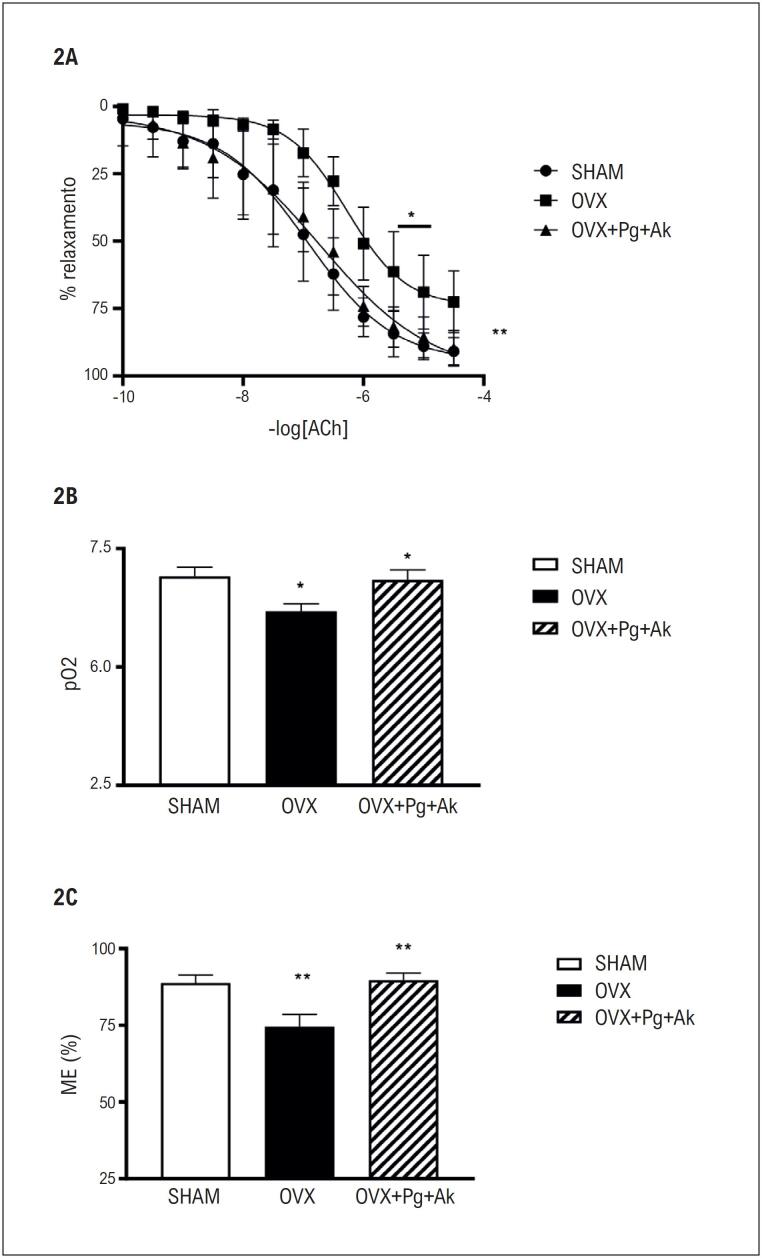
Estudo da função endotelial em anéis de aorta isolados dos grupos Sham, OVX e OVX + Pg + Ak. Curvas concentração-efeito cumulativas foram realizadas para a acetilcolina em aortas pré-contraídas com fenilefrina. Cada ponto representa a média e o erro padrão da média (EP) dos dados obtidos a partir de determinações independentes. 2A e 2B; * Indicam diferença (p < 0,05) nos valores de pD2 entre as aortas dos grupos Sham vs OVX e OVX vs OVX + Pg + Ak; 2A e 2C ** Indicam diferença (p < 0,01) nos valores de Emax entre Sham vs OVX e OVX vs OVX + Pg + Ak; OVX: ratas ovariectomizadas; OVX + Pg + Ak: ratas ovariectomizadas tratadas com extrato padronizado de Panax ginseng + Angelica keiskei.

**Figura 3:**
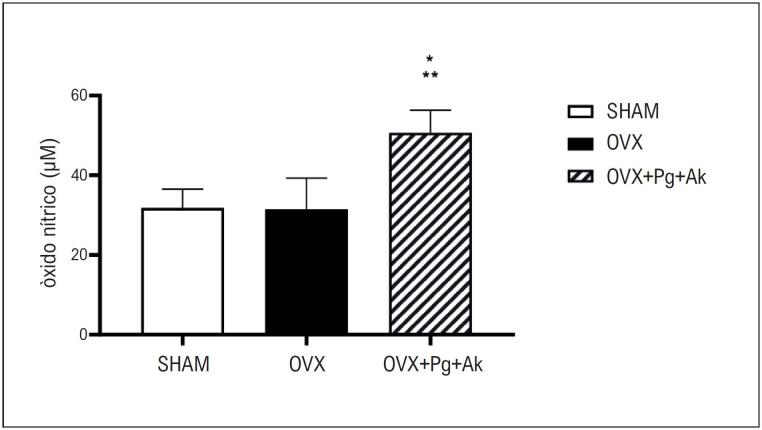
Níveis de óxido nítrico (NO) no sangue de ratas ovariectomizadas (OVX) em comparação com os grupos OVX + Pg + Ak e Sham. *Indica diferença (p < 0,05) nos níveis de NO em OVX + Pg + Ak vs. OVX. Indica diferença (p < 0,05) nos níveis de NO no grupo OVX + Pg + Ak vs. Sham. OVX: ratas ovariectomizadas; OVX + Pg + Ak: ratas ovariectomizadas tratadas com extrato padronizado de Panax ginseng + Angelica keiskei.

O relaxamento dependente do endotélio induzido por acetilcolina está prejudicado nos anéis aórticos de ratas OVX (pD2: 6,17 ± 0,16, n = 8; EM: 72,46 ± 4,03%, n = 8), em comparação ao grupo controle Sham (pD2: 6,91 ± 0,19, n = 8; EM: 90,84 ± 1,78%, n = 8). O tratamento crônico com *Panax ginseng* + *Angelica keiskei* (Pg + Ak) melhorou a VDE nos anéis aórticos de ratas OVX (pD2: 6,83 ± 0,20, n = 8; EM: 89,75 ± 2,29%, n = 8), em comparação ao grupo OVX (pD2: 6,17 ± 0,16, n = 8; EM: 72,46 ± 4,03%, n = 8), sem diferença entre OVX + Pg + Ak e Sham ( Figuras 2A, 2B e 2C ). Além disso, o tratamento crônico com *Panax ginseng* + *Angelica keiskei* (Pg + Ak) induziu aumento nos níveis sanguíneos de NO em ratas OVX (OVX + Pg + Ak: 50,69 ± 2,82 *µ* M, n = 5), em comparação ao grupo OVX (31,45 ± 2,98 *µ* M, n = 5) e ao grupo Sham (31,79 ± 2,11 *µ* M, n = 5) ( Figura 3 ).

## Discussão

Neste estudo, observamos que o tratamento com *Panax ginseng* e *Angelica keiskei* por duas semanas resultou em redução da pressão arterial, melhora da função endotelial e aumento nos níveis circulantes de NO. A ovariectomia induziu efetivamente disfunção endotelial e elevação da pressão arterial.

O *Panax ginseng* é uma planta antiga com usos medicinais enraizada na medicina tradicional chinesa. Seus efeitos anti-hipertensivos, especialmente no sistema cardiovascular, são atribuídos, em parte, ao aumento de NO circulante derivado do endotélio. Essa planta ativa a enzima eNOS (óxido nítrico sintase endotelial), que converte a L-arginina em L-citrulina e NO, levando à ativação da enzima guanilato ciclase solúvel nas células musculares lisas, induzindo vasorrelaxamento e, consequentemente, redução da pressão arterial.^19 , 42^ Em nosso estudo, observamos esse efeito em ratas OVX. Os principais agentes responsáveis por esse mecanismo são os ginsenosídeos Rb1, Rg1 e Rg3, que estimulam a produção endotelial de NO.^36 , 43^

Além disso, o Rg3 é reconhecido como o mais potente vasodilatador entre os ginsenosídeos. Sua ação envolve a inibição do tônus da musculatura lisa vascular por meio da prevenção do influxo de Ca²^+^ e estímulo ao efluxo de K^+^.^44^ Este estudo confirmou os efeitos hipotensores do *Panax ginseng* em ratas.^44^ O ginsenosídeo Rg3 também promove o aumento da expressão de eNOS, intensificando a produção de NO e levando à vasodilatação.^45^

Em relação aos extratos de *Angelica keiskei* , estudos indicam que eles exercem efeitos protetores vasculares contra a vasoconstrição induzida por fenilefrina por meio de mecanismos mediados por NO e fator relaxante derivado do endotélio.^46^ Pesquisas também demonstraram que compostos como Xantoangelol (XAG) e 4-hidroxidericina (4-HD) influenciam a função plaquetária, podendo prevenir doenças trombóticas.^47 , 48^ A capacidade de *Angelica keiskei* em reduzir o tempo de sangramento na cauda de camundongos sugere um efeito sobre a agregação plaquetária *in vivo* .^47^

Uma revisão concluiu que o XAG pode inibir diretamente as funções da musculatura lisa ao reduzir o cálcio intracelular livre (Ca²^+^), enquanto a 4-hidroxidericina suprime a elevação de Ca²^+^induzida por fenilefrina.^29^ Flavonoides presentes na *Angelica keiskei* possuem uma estrutura complexa que inibe a enzima conversora de angiotensina, uma enzima-chave na regulação da pressão arterial. Esses compostos flavonoides atuam como agentes anti-hipertensivos, reduzem o estresse e inibem a atividade oxidativa da enzima conversora de angiotensina.^49^

Embora alguns estudos tenham examinado os efeitos biológicos combinados de *Panax ginseng* e *Angelica keiskei* , nenhum deles focou em seus efeitos cardiovasculares. Muitos dos componentes individuais dessas plantas são conhecidos por beneficiar a saúde cardiovascular. Assim, a combinação desses extratos parece uma opção promissora para o tratamento de doenças cardiometabólicas.

Nossos achados sugerem que a combinação de *Panax ginseng* e *Angelica keiskei* pode promover melhorias de longo prazo na função vascular. O *Panax ginseng* , por meio de seus ginsenosídeos, ativa vias-chave de vasodilatação, enquanto a *Angelica keiskei* demonstrou diversos efeitos benéficos em distúrbios metabólicos e doenças associadas à inflamação, sendo os xantoangelóis seus principais compostos ativos.

Uma limitação do estudo é que o grupo Sham foi submetido apenas à simulação cirúrgica sem receber solução salina, que foi utilizada como veículo para os tratamentos com plantas. Portanto, o grupo Sham não recebeu nenhum tratamento, o que pode limitar a interpretação das diferenças observadas entre os grupos tratados com as plantas e o grupo Sham. Além disso, os tratamentos com as plantas foram administrados por injeção intraperitoneal, o que, embora eficaz para o protocolo experimental, não reproduz totalmente a administração oral, que seria mais representativa do uso humano no dia a dia. Essa diferença na via de administração pode afetar a farmacocinética e a aplicabilidade geral dos achados para o tratamento em humanos.

Neste estudo, observamos melhora do relaxamento dependente do endotélio nos anéis aórticos do grupo OVX + Pg + Ak, sugerindo que a disfunção endotelial induzida pela ovariectomia pode ser revertida por meio do tratamento com *Panax ginseng* e *Angelica keiskei* . Nossos resultados indicam que esse tratamento combinado melhora a função endotelial e eleva os níveis circulantes de NO, o que provavelmente contribui para a redução observada da pressão arterial nas ratas OVX.

## Conclusão

Em conjunto, nossos resultados mostram que a ovariectomia pode induzir elevação da pressão arterial sistólica e disfunção endotelial, as quais foram reduzidas após duas semanas de tratamento com a associação de *Panax ginseng* e *Angelica keiskei* .

Disponibilidade de Dados

Os conteúdos subjacentes ao texto da pesquisa estão contidos no manuscrito.

## *Material suplementar

Para informação adicional, por favor, clique aqui .
