# Supplementary material for: Tratamento Crônico com Panax ginseng e Angelica keiskei Reduz a Pressão Arterial e Melhora a Função Endotelial em Ratas Ovariectomizadas
Source: Arq Bras Cardiol. 2025 Aug 20;122(8):e20240685. [Article in Portuguese] doi: 10.36660/abc.20240685 (PMC12671595; doi:10.36660/abc.20240685)

SUPPLEMENTARY MATERIAL

Image 1A: Uterus of a non-ovariectomized rat, with preserved ovaries. 1B: Uterus of an ovariectomized rat, without the ovaries.

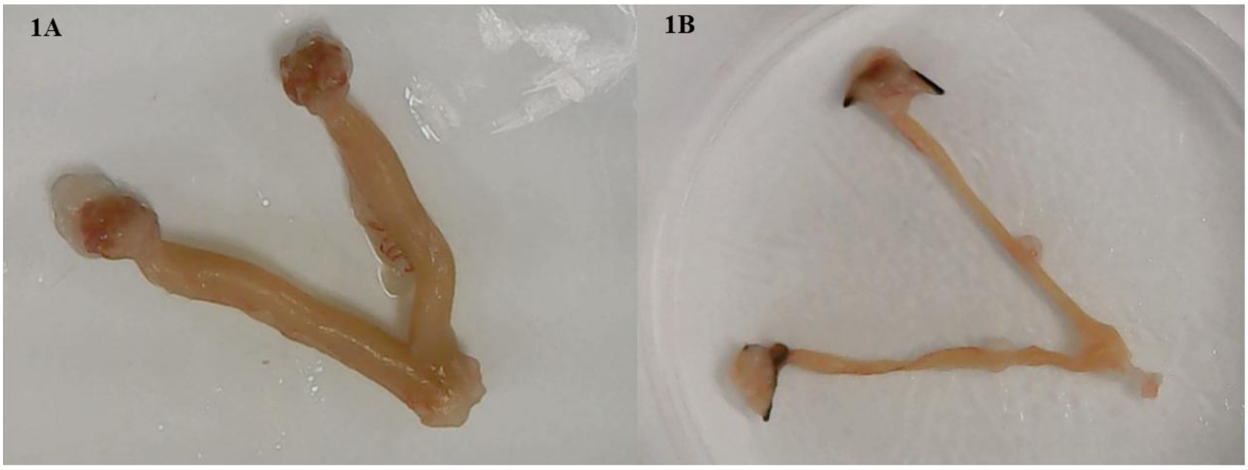

Supplement: Supplementary file 1 [file 0066-782X-abc-122-08-e20240685-Suppl01.pdf]
