# Supplementary material for: Tratamento Crônico com Panax ginseng e Angelica keiskei Reduz a Pressão Arterial e Melhora a Função Endotelial em Ratas Ovariectomizadas
Source: Arq Bras Cardiol. 2025 Aug 20;122(8):e20240685. [Article in Portuguese] doi: 10.36660/abc.20240685 (PMC12671595; doi:10.36660/abc.20240685)
Supplement: Translation

# Translation: Chronic Treatment with *Panax ginseng* and *Angelica keiskei* Decreases Blood Pressure and Improves Endothelial Function in Ovariectomized Rats

**DOI:** 10.36660/abc.20240685i

**Authors:** Nayara Formenton da Silva, Luis Henrique Oliveira de Moraes, Camila Pereira Sabadini, Rita Cristina Cotta Alcântara, Patrícia Corrêa Dias, Gerson Jhonatan Rodrigues

**Affiliations:** 1 São Carlos SP Brazil Universidade Federal de São Carlos (UFSCar), São Carlos, SP – Brazil

**Keywords:** Ovariectomy, Menopause, Hypertension, Herbal Medicine, Panax

## Abstract

**Background:**

Menopause is a natural physiological process that can impact various systems and organs, often leading to increased blood pressure. The combination of *Panax ginseng* and *Angelica keiskei* (Pg + Ak) is a formulation known to improve vascular function.

**Objective:**

This study aimed to evaluate the *in vivo* effects of these combined plants on cardiovascular parameters in female rats experiencing menopause induced by ovariectomy.

**Methods:**

Twenty-four 70-day-old female Wistar rats were randomly assigned to three groups: sham-operated (Sham), ovariectomized (OVX), and ovariectomized with Pg + Ak treatment (OVX + Pg + Ak). The rats in the Pg + Ak treatment group received an intraperitoneal injection of 100 mg/kg/day for two weeks. Blood pressure was measured using tail plethysmography, and endothelial function was assessed through vascular reactivity tests.

**Results:**

At the end of the treatment, mean blood pressure in the SH group was lower than in the control group (OVX), and the Pg + Ak treatment improved the endothelial dysfunction caused by ovariectomy. This was evident in the restored endothelium-dependent vasodilation in the aortic rings of the OVX rats treated with Pg + Ak compared to untreated OVX rats.

**Conclusions:**

Our findings suggest that the Pg + Ak combination effectively reduces blood pressure and reverses endothelial dysfunction in ovariectomized rats.

## Introduction

Menopause is a natural physiological process marked by the permanent cessation of menstruation for 12 months, typically related to age-related estrogen deficiency and not linked to pathology. The median age for menopause is around 51 years. Although most women experience vasomotor symptoms, menopause also affects various body systems, including the urogenital, psychogenic, and cardiovascular systems.^1 - 4^A decrease in estrogen levels disrupts the hypothalamic-pituitary-ovarian axis, leading to the failure of endometrial development, which results in irregular menstrual cycles that ultimately stop altogether.^5^

Estrogen plays a critical role in maintaining endothelial function by increasing nitric oxide (NO) synthesis in the vascular endothelium. NO diffuses into vascular smooth muscle cells, causing relaxation – a process known as endothelium-dependent vasodilation (EDV). Estrogen also helps maintain endothelial function by reducing the synthesis of endothelin-1, a potent vasoconstrictor, in endothelial cells. During menopause, EDV decreases, while endothelin-1 synthesis rises, both contributing to increased vasoconstriction.^6^

Additionally, estrogen deficiency can impact various organs and systems, including the cardiovascular system, increasing the risk of diseases like hypertension. According to Global Burden of Disease data, hypertension is the leading deadly risk factor for women worldwide.^7^The incidence of cardiovascular disease in postmenopausal women is two to six times higher than in premenopausal women of the same age group.^8^To address these symptoms, hormone replacement therapy (HRT) is often recommended as a means of mitigating estrogen deficiency, thereby enhancing quality of life across physical, emotional, and sexual health dimensions.^9^However, due to the known side effects and potential contraindications of HRT, alternative botanical therapies are increasingly recognized as beneficial for this population.^10^

One of the most widely used ginseng species today is *Panax ginseng* , primarily grown in Korea and China, which accounts for a significant portion of global ginseng production.^11^The primary active compounds in *Panax ginseng* are ginsenosides, which are triterpene saponins. Much research on the pharmacological and medicinal properties of *Panax ginseng* focuses on ginsenosides, including Rb1, Rg1, Rg3, Re, and Rd.^12^

Ginseng has demonstrated several biological activities, including anticancer properties,^13 , 14^antidiabetic effects,^15 , 16^and cardiovascular benefits, such as aiding in hypertension treatment.^17 - 19^Ginsenosides Rb1 and Rg1 exhibit anti-inflammatory effects by inhibiting antioxidant production and promoting NO synthesis.^20 , 21^Additionally, these ginsenosides have antioxidant properties, preventing reactive oxygen species (ROS) production through NO stimulation. Ginsenoside Rb1 and other ginsenosides also counter endothelial dysfunction by activating the SIRT1/AMPK signaling pathway, inhibiting ROS production, activating estrogen receptor beta (ER-β), and upregulating superoxide dismutase (SOD).^23 , 24^

*Angelica keiskei* , commonly known as ashitaba or "tomorrow's leaf," is a hardy perennial plant native to Japan's Pacific coast.^25^This plant has gained attention for its medicinal properties, including antioxidative, anti-inflammatory, hypoglycemic, antimicrobial, antitumor, hypotensive, antifibrotic, laxative, stimulant, and galactagogue effects.^25 - 29^While the specific active components of *Angelica keiskei* remain unidentified, over 100 compounds have been isolated from it, including chalcones, coumarins, and flavanones.^29^

A product known as Mitochondrin® combines *Panax ginseng* and *Angelica keiskei* , featuring a triple standardization of 8% chalcones, 10% flavanones, and 0.9% ginsenosides Rb1, Rg1, and Rg3, providing a multi-targeted action on several epigenetic mechanisms. These components enhance mitochondrial biogenesis, vital for energy production and healthy aging, while promoting caloric expenditure, reducing fat storage, and minimizing inflammation through antioxidant effects.^30^

These plants are widely used in traditional Chinese medicine for their physiological benefits, which include reducing free radicals,^31 , 32^controlling inflammation,^33 , 34^regulating coagulation,^35^promoting NO production,^36^enhancing glucose metabolism,^37^and reducing mitochondrial dysfunction.^23 , 38^Complementary therapies are increasingly prominent as a safe option for managing menopausal symptoms. In this study, we investigate whether the unique combination of *Panax ginseng* and *Angelica keiskei* can improve vascular parameters in ovariectomized rats.

## Material and Methods

### Animals and experimental design

Twenty-four female Wistar rats with 200 g to 250 g were divided randomly (each animal was assigned a number, and groups were determined using a random number generator, preventing selection bias) into three groups: ovariectomized (OVX) (n = 8), ovariectomized rats treated with standardized extract of *Panax ginseng* + *Angelica keiskei* (OVX + Pg + Ak) (n = 8), and sham (Sham) (n = 8). All the procedures with rats that will be described in this work were conducted after one week of acclimatization. The animals were kept in the light-dark cycle with food and water *ad libitum* until the experiment day. All animals were weighed every two weeks.

### Menopause induction

All animals in the OVX groups underwent ovariectomy in the 12th week of life, due to the good response to surgery evidenced in the literature.^38^The technique used followed the Zarrow protocol,^39^and anesthesia was performed with an association of 13 mg/Kg of xylazine and 33 mg/Kg of ketamine base by parenteral administration (intramuscular). First, a small bilateral incision (1.0-1.5 cm) was made through the skin and muscle layer using scissors and tweezers, approximately 1 cm below the last rib, perpendicular to the animal's body.

For the procedure, the peritoneal cavity was opened, the ovaries exposed and removed, and a nylon suture ligated just below the fimbria. After removing the ovaries, an incision was made in the musculature and skin with the nylon thread. The animals in the sham groups underwent the same surgical procedure, had the ovaries externalized to the abdominal cavity and returned, without removing them. After the surgery, a period of two weeks of recovery was respected to start the procedures with the standardized extract administration protocol.

### Blood pressure measurement

Throughout the treatment, systolic blood pressure was measured in the animals using tail-cuff plethysmography in non-anesthetized animals, prior to menopause induction, six weeks after induction, and following treatment. The rats were placed in a restraint and acclimatization apparatus within a quiet and calm environment for one hour. This procedure was repeated a few times before the analysis, with the concern of making them familiar with the test.

The plethysmograph has a rubberized cuff and a photoelectric pulse sensor that was placed around the animal's tail (model Power Lab 8/35, AD Instruments, Pty Ltda, Colorado Springs, CO). Three consecutive measurements were performed, considering the arithmetic mean of these results as the pressure value.

### Administration of standardized extracts of *Panax ginseng* + *Angelica keiskei*

The animals in the groups that received the standardized extract treatment were treated with 100 mg/kg/day for two weeks and the standardized extract was administered intraperitoneally.^23^All animals were weighed so that the dose of the standardized extract was adequate. The animals in the groups that did not receive treatment with the standardized extract were handled with the same frequency as the treated ones. The standardized extracts of *Panax ginseng* + *Angelica keiskei* association (1:1). These standardized extracts make up the formulation of a product registered under the name Mitochondrin^®^. This product has a triple standardization of 8% chalcones, 10% flavanones, and 0.9% ginsenosides Rb1, Rg1, Rg3. The extracts were solubilized in physiological saline (sodium chloride 0.9%), just before treatment.

### Euthanasia and biological material collect

The animal, anesthetized with isoflurane, were sacrificed by decapitation and some tissues were collected for further analysis. The aortic thoracic artery was removed, isolated and cut into rings of approximately 4 mm in length and kept in Krebs solution to vascular reactivity experiment. The uterus was collected and photographed for further analysis. The success of the surgery was verified by the uterine atrophy observed in the ovariectomized groups when compared to the sham group, as can be verified at the Central Illustration and supplementary material ( Image 1A ). Blood was collected in falcon tubes shortly after euthanasia for subsequent serum aliquots for biochemical tests.

### Vascular reactivity

The aortic rings dissected after euthanasia were placed in an isolated organ bath containing 5 mL of Krebs solution at 37 °C, pH 7.4, continuously bubbled with 95% O_2_and 5% CO_2_in an isometric myograph (Mulvany-Halpern-model 610 DMT-USA, Marietta, GA) and recorded by a PowerLab8/SP data acquisition system (ADInstruments Pty Ltd., Colorado Springs, CO).

Firstly, the aortic rings were submitted to a tension of 1.5 g for thirty minutes to allow for stabilization. Then, the endothelial integrity was assessed using the EC_50_of phenylephrine (0.1 *µ* mol/L) to contract the vessel, followed by the relaxation induced by 1 *µ* mol/L acetylcholine. Rings were discarded when the relaxation was lower than 80 %, assuming what has been standardized for the hypertensive endothelial dysfunctional rats.^40^

The intact aortic rings were submitted to a second contraction with 0.1 *µ* mol/L phenylephrine and then were constructed concentration-effect curves to acetylcholine (0.1 nmol/L to 0.1 mmol/L). The potency (pD2) and the maximal relaxant effect were evaluated.

### Nitric oxide measurement

For nitric oxide (NO) measurement levels, serum concentration of NO stable product was measured, nitrite (NO_2_^-^) and nitrate (NO_3_^-^), known as NOx, as described previously,^41^by using NO Analyzer 280i (Sievers, Boulder, CO, USA).

### Statistical analysis

The statistical analyses of the results were performed using GraphPad Prism software (version 8.0.1). Data distribution was assessed for normality using the Shapiro-Wilk test. Since the data followed a normal distribution, they were presented as mean ± standard error of the mean (SEM). Comparisons among all groups were made using one-way ANOVA with the Newman-Keuls post-test. A significance level of 95% (α = 0.05) was adopted for all statistical analyses, ensuring that the probability of Type I error remained within an acceptable threshold for robust and reliable inference.

## Results

Ovariectomy induced elevation in systolic blood pressure, as can be observed in the group OVX (141.62 ± 6.0 mmHg, n = 8) and OVX + Pg + Ak before the treatment (143.77 ± 5.79 mmHg, n = 8) compared to Sham (123.77 ± 4.16 mmHg, n = 8). Two-week treatment with association of *Panax ginseng* + *Angelica keiskei* induced a decrease in systolic blood pressure in OVX rats (OVX + Pg + Ak: 118.22 ± 4.8 mmHg, n = 8), compared to the OVX group with no treatment (139.12 ± 7.1 mmHg, n = 8) ( Figure 1 ).

**Figure 1:** Systolic blood pressure before and after two-week treatment. Each dot represents the mean and mean standard error (SEM) of data obtained from independent determinations.; *difference (p < 0.05) in systolic blood pressure values in Sham vs. OVX and Sham vs. OVX + Pg + Ak before Pg + Ak treatment (time = 0). **Indicates the difference (p < 0.05) in values between systolic blood pressure from OVX vs OVX+ Pg + Ak after two-week treatment with Pg + Ak (time = 2); OVX: ovariectomized rats; OVX + Pg + Ak: ovariectomized rats treated with standardized extract of Panax ginseng + Angelica keiskei.

The endothelium-dependent relaxation induced by acetylcholine is impaired in aortic rings in OVX rats (pD2: 6.17 ± 0.16, n = 8; ME: 72.46 ± 4.03%, n = 8), compared to control Sham (pD2: 6.91 ± 0.19, n = 8; ME: 90.84 ± 1.78%, n = 8). The chronic treatment with *Panax ginseng* + *Angelica keiskei* (Pg + Ak) improved the endothelium-dependent vasodilation in aortic rings in OVX rats (pD2: 6.83 ± 0.20, n = 8; ME: 89.75 ± 2.29%, n = 8) compared to OVX (pD2: 6.17 ± 0.16, n = 8; ME: 72.46 ± 4.03%, n = 8), with no difference between OVX + Pg + Ak and Sham ( Figures 2A, 2B and 2C ). In addition, chronic treatment with *Panax ginseng* + *Angelica keiskei* (Pg + Ak) induced an increase in NO blood levels in OVX rats (OVX + Pg + Ak: 50.69 ± 2.82 *µ* M, n = 5) compared to OVX (31.45 ± 2.98 *µ* M, n = 5) and with Sham (31.79 ± 2.11 *µ* M, n = 5) ( Figure 3 ).

**Figure 2:** Study of the endothelium function in isolated aortic rings vessels from Sham, OVX and OVX + Pg + Ak rats. Cumulative concentration-effect curves performed for the acetylcholine in aortas pre-contracted with phenylephrine. Each dot represents the mean and mean standard error (SEM) of data obtained from independent determinations. 2A and 2B * Indicates the difference (p < 0.05) in pD2 values between aortas from Sham vs OVX and OVX vs OVX + Pg + Ak. 2A and 2C ** Indicates the difference (p < 0.01) in Emax values between Sham vs OVX and OVX vs OVX + Pg + Ak; OVX: ovariectomized rats; OVX + Pg + Ak: ovariectomized rats treated with standardized extract of Panax ginseng + Angelica keiskei.

**Figure 3:** Nitric oxide blood levels from ovariectomized (OVX) rats compared to OVX + Pg + Ak and with Sham. *Indicates the difference (p < 0.05) in NO levels in OVX + Pg + Ak vs. OVX. **Indicates the difference (p<0.05) in NO levels in OVX + Pg + Ak vs. SHAM; OVX: ovariectomized rats; OVX + Pg + Ak: ovariectomized rats treated with standardized extract of Panax ginseng + Angelica keiskei.

## Discussion

In this study, we found that treatment with *Panax ginseng* and *Angelica keiskei* for two weeks resulted in a reduction in blood pressure, improved endothelial function, and increased circulating NO levels. Ovariectomy effectively induced endothelial dysfunction and blood pressure elevation.

*Panax ginseng* is an ancient plant with medicinal uses rooted in traditional Chinese medicine. Its antihypertensive effects, particularly on the cardiovascular system, are partly attributed to increased circulating NO derived from the endothelium. This plant activates the enzyme eNOS (endothelial nitric oxide synthase), which converts L-arginine into L-citrulline and NO, leading to activation of the soluble guanylate cyclase enzyme in smooth muscle, inducing vasorelaxation and, consequently, a decrease in blood pressure.^19 , 42^In our study, we observed this effect in OVX rats. The primary agents responsible for this mechanism are the ginsenosides Rb1, Rg1, and Rg3, which stimulate endothelial NO production.^36 , 43^

Additionally, Rg3 is recognized as the most potent vasodilator among the ginsenosides. Its action involves inhibiting vascular smooth muscle tone by preventing Ca²^+^influx and stimulating K^+^efflux.^44^This study confirmed the hypotensive effects of *Panax ginseng* in rats.^44^Ginsenoside Rg3 also promotes increased eNOS expression, enhancing NO production and leading to vasorelaxation.^45^

Regarding *Angelica keiskei* extracts, studies indicate that they exert vascular protective effects against phenylephrine-induced vasoconstriction through NO and endothelium-derived relaxing factor (EDRF) mechanisms.^46^Research has also shown that compounds such as Xanthoangelol (XAG) and 4-hydroxyderricin (4-HD) influence platelet function, potentially preventing thrombotic diseases.^47 , 48^The ability of *Angelica keiskei* to reduce tail bleeding duration in mice suggests an effect on platelet aggregation *in vivo* .^47^

A review concluded that XAG can directly inhibit smooth muscle functions by reducing intracellular free calcium (Ca²^+^), while 4-hydroxyderricin suppresses phenylephrine-induced Ca²^+^elevation.^29^Flavonoids in *Angelica keiskei* possess a complex structure that inhibits angiotensin-converting enzyme, a key enzyme in regulating blood pressure. These flavonoid compounds serve as antihypertensive agents, reduce stress, and inhibit angiotensin-converting enzyme oxidative activity.^49^

Although a few studies have examined the combined biological effects of *Panax ginseng* and *Angelica keiskei* , none has focused on their cardiovascular effects. Many individual components of these plants are known to benefit cardiovascular health. Thus, combining these extracts appears to be a promising option for treating cardiometabolic diseases.

Our findings suggest that the combination of *Panax ginseng* and *Angelica keiskei* may yield long-term improvements in vascular function. *Panax ginseng* , with its ginsenosides, activates key vasorelaxation pathways, while *Angelica keiskei* has demonstrated various beneficial effects in metabolic disorders and other diseases associated with inflammation, with xanthoangelols being the primary active compounds.

One limitation of the study is that the Sham group underwent only the surgical simulation without receiving saline, which was used as the vehicle for the plant treatments. Therefore, the Sham group did not receive any treatment, and this may limit the interpretation of the differences observed between the plant-treated groups and the Sham group. Additionally, the plant treatments were administered via intraperitoneal injection, which, although effective for the experimental protocol, does not fully replicate the oral administration that would be more representative of human usage in daily life. This difference in administration route could affect the pharmacokinetics and overall applicability of the findings to human treatment.

In this study, we observed improved endothelium-dependent relaxation in aortic rings from the OVX + Pg + Ak group, suggesting that ovariectomy-induced endothelial dysfunction can be reversed through treatment with *Panax ginseng* and *Angelica keiskei* . Our results indicate that this combined treatment enhances endothelial function and raises circulating NO levels, which is likely to contribute to the observed reduction in blood pressure in OVX rats.

## Conclusion

Taken together, our results show that ovariectomy can induce elevation of systolic blood pressure and endothelial dysfunction, which were reduced by two-week treatment with the *Panax ginseng* and *Angelica keiskei* association.

Data Availability

The underlying content of the research text is contained within the manuscript.

## *Supplemental Materials

For additional information, please click here .
